# Impact of Political Leaning on COVID-19 Vaccine Hesitancy: A Network-Based Multiple Mediation Analysis

**DOI:** 10.7759/cureus.43232

**Published:** 2023-08-09

**Authors:** Farrokh Alemi, Kyung Hee Lee

**Affiliations:** 1 Health Administration and Policy, George Mason University, Fairfax, USA; 2 Recreation, Parks, and Leisure Service, Central Michigan University, Mount Pleasant, USA

**Keywords:** covid-19, lasso, multiple mediation analysis, social determinants, political leaning, covid-19 vaccination

## Abstract

Prior studies have shown that political affiliation affected COVID-19 vaccine hesitancy. This study re-examined the data to see if these findings hold after controlling for alternative explanations. The dependent variable in the study was COVID-19 vaccination rates in 3,109 counties in the United States as of April 2022. The study examined 36 possible alternative explanations for vaccine hesitancy, including demographic, social, economic, environmental, and medical variables known to affect vaccine hesitancy. County-level political affiliation was measured as a percent of voters in the county who were affiliated with Democratic or Republican political parties. Data were analyzed using a temporally constrained multiple mediation network, which allowed for the identification of both direct and indirect predictors of vaccination rates. Despite controlling for alternative explanations of hesitancy, there was a statistically significant relationship between the percentage of Republican supporters and rates of vaccine hesitancy. The higher the Republican affiliation, the lower the vaccination rates. It is possible that the Republican Party has played an organizing role in encouraging vaccine hesitancy and patient harm.

## Editorial

Introduction

Political leaning is frequently linked to particular values and ideological frameworks, which can shape people's attitudes and beliefs on a broad range of topics, including public health and vaccination [1-3]. For instance, individuals with a more conservative leaning may prioritize personal freedoms and government intervention limitations, which can influence their views on mandated vaccinations [4]. Conversely, those with a more progressive leaning may prioritize collective well-being and consider vaccination a public health responsibility [5].

Several recently published studies found a relationship between political preference and COVID-19 vaccination hesitancy. Albrecht found that Republican-leaning counties (as measured by the percentage of the residents voting for Trump) experienced lower vaccination rates for COVID-19, higher COVID-19 cases, and higher death rates [6]. His subsequent research, using the most recent data, found that the relationship between political views, vaccination rates, and COVID-19 death rates became even stronger over time [7]. Sargent and colleagues also found that vaccination is based on political leaning [8]. More recently, Pallathadka and colleagues identified six statistically significant predictors of COVID-19 vaccination rates at the county level in the contiguous United States [9]. Specifically, the percentage of Republican voters and the percentage of the Black population were negatively correlated with vaccination rates, while four remaining predictors, the percentage of the population with a college degree, National Risk Index (NRI) score, the percentage of the population with broadband access, and the number of health facilities per 10,000 population, were positively correlated with vaccination rates.

Since political leaning is affected by the same set of variables (e.g., race, gender, and education) as vaccine hesitancy, it is not clear if the observed differences are due to confounding in observational data. It is possible that the observed relationship between political leaning and vaccination hesitancy disappears if one statistically controls for other explanations of vaccine hesitancy. The main purpose of our study is to re-examine the effect of political leaning on vaccination rates, after controlling for a larger set of demographics, social, medical, access, and environmental determinants of vaccine hesitancy.

Materials and methods

Data were collected from multiple secondary sources, including the U.S. Census, Centers for Disease Control and Prevention, Wayback Internet Machine (https://archive.org/web/), U.S. Census Bureau (American Community Survey (ACS), Small Area Income and Poverty Estimates (SAIPE), and population estimates and projection), U.S. Environmental Protection Agency (EJScreen data), U.S. Department of Agriculture, Environmental Systems Research Institute (% political affiliation), and the National Center for Health Statistics.

In the analysis, the dependent variable was COVID-19 vaccination rates in 3,109 counties in the United States as of April 2022. The independent variables included a total of 34 demographic, socioeconomic, environmental, access, and medical characteristics of these counties. We measured political affiliation by the percentage of county residents affiliated with Democratic or Republican political parties. All independent variables were measured at two timeframes to allow interpretation of the directionality of associated variables.

**Figure 1:**
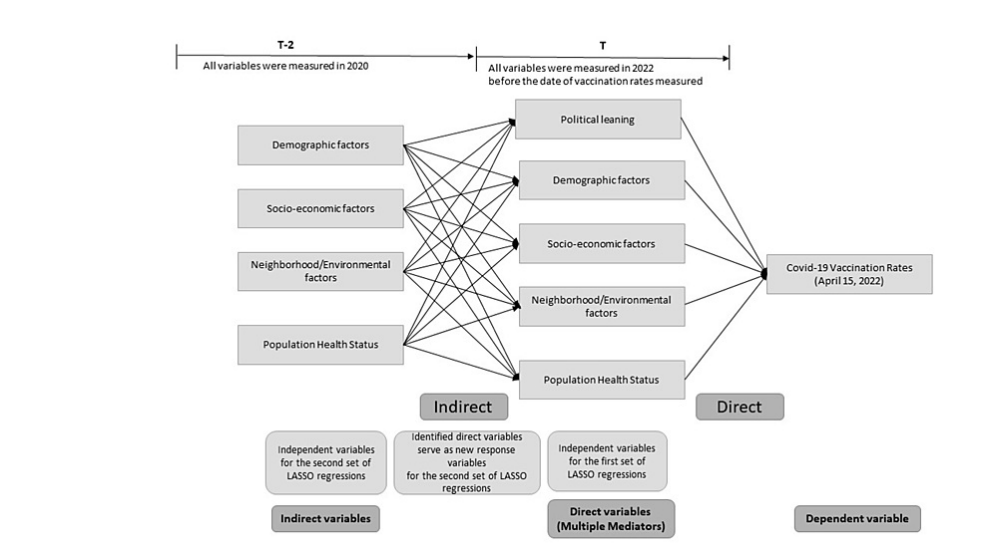
Analytical framework

Data were analyzed using a chain of temporally constrained LASSO (least absolute shrinkage and selection operator) regressions. This methodology allows for the identification of both direct and indirect predictors of vaccination rates. The first regression identifies the direct predictors of vaccination rates. Subsequently, the direct predictors are regressed on variables preceding in time to identify the indirect predictors of vaccination rates (Figure 1). The methodology is similar to the methods used by the previous study [6] but relies on a large set of variables and utilizes time constraints to eliminate the possibility of reverse causal findings or mediator-mediator interaction effects that could distort the relationship between the direct causes of vaccination.

Results

Table 1 presents the results of chained LASSO for identifying direct and indirect predictors of COVID-19 vaccination rates, after controlling mediator-mediator interactions. The first set of LASSO regressions identified direct predictors of COVID-19 vaccination rates. Only variables shown nine out of 10 times through repeated LASSO were included in Table 1. The direct variables underwent ordinary least squares (OLS) regression utilizing indirect variables measured in 2020, which were identified through subsequent LASSO sets. A total of 46.8% of variations in vaccination rates were explained by direct variables. Both Republican (regression coefficient: -0.24) and Democratic affiliation (regression coefficient: 0.16) were direct predictors of COVID-19 vaccination rates.

**Table 1:** Results of chained LASSO for identifying direct and indirect predictors of vaccination rates Note: Please be aware that despite being included as independent variables in the regression analysis, none of the following variables demonstrated statistical significance in any of the regression models: % of Hispanics, % of Native Hawaiians or other Pacific Islanders, % open space, % male, % driving alone to work, % injury/death, length of commute driving, % residential segregation Black versus White, % residential segregation non-White vs. White, and income equality. LASSO: least absolute shrinkage and selection operator.

	Dependent variable in regression															
Prior year independent variables (below)	Complete-series vaccination 2022	Republican affiliation	Democratic affiliation	Asian	American Indian or Alaska Native	Life expectancy	Disability	Older than 65	Some college	Premature mortality	Median household income	Children in poverty	Unemployed	Not proficient in English	High school completion	# of vaccination providers
# of vaccination providers	0.07	.	.	.	.	.	.	.	.	.	.	.	.	.	.	.
% Not proficient in English	0.3	.	.	.	.	.	.	.	.	.	.	.	.	0.76	.	.
% American Indian or Alaska Native	0.4	.	.	.	1.02	.	.	.	.	.	.	.	.	.	.	.
% Asian	0.17	.	.	0.95	.	.	.	.	.	.	-0.1	.	.	.	.	.
% Below 18 years of age	.	0.36	-0.35	.	.	.	.	.	.	.	.	.	.	.	.	.
% Children in poverty	-0.2	.	.	.	.	.	.	.	.	.	.	0.71	.	.	.	.
% Children in single-parent households	.	.	.	.	.	.	.	.	.	.	.	-0.1	.	.	.	.
% Disability population	-0.2	.	.	.	.	.	0.33	.	.	.	.	.	.	.	.	.
% Unemployed	0.16	-0.26	0.3	.	.	.	.	.	.	.	-0.01	.	1.05	.	.	.
% High school completion	0.21	.	.	.	.	.	.	.	.	.	.	.	.	.	0.66	.
% Homeownership	.	0.12	-0.1	.	.	.	.	.	.	.	.	.	.	.	.	.
% Life expectancy	0.24	.	.	.	.	0.4	.	.	.	.	.	.	.	.	.	.
% Non-Hispanic White	.	0.18	.	.	.	.	.	.	.	.	.	.	.	.	.	.
% Older than 65	0.39	0.44	-0.25	.	.	.	.	0.97	.	.	.	.	.	.	.	.
% Premature mortality	-0.17	.	.	.	.	.	.	.	.	0.57	.	.	.	.	.	.
% Democratic affiliation	0.16	.	.	.	.	.	.	.	.	.	.	.	.	.	.	.
% Republican affiliation	-0.24	.	.	.	.	.	.	.	.	.	.	.	.	.	.	.
% Ruralized area	.	0.19	-0.12	.	.	.	.	.	.	.	.	.	.	.	.	.
% Severe housing cost burden	.	-0.18	0.1	.	.	.	.	.	.	.	.	.	.	.	.	.
% Some college	0.26	.	.	.	.	.	.	.	0.54	.	.	.	.	.	.	.
% Traffic volume	.	-0.2	0.29	.	.	.	.	.	.	.	.	.	0.2	.	.	.
Median household income	0.08	-0.07	0.38	.	.	.	.	.	.	.	0.87	0.16	.	.	.	.
% Non-Hispanic Black	.	.	0.27	.	.	.	.	.	.	.	.	.	.	.	.	.
% Female	.	.	0.24	.	.	.	.	.	.	.	.	.	.	.	.	.
Poverty rate	.	.	0.4	.	.	.	.	.	.	.	0.15	.	.	.	.	.
Total population	.	.	.	.	.	.	.	.	.	.	.	.	.	.	.	1.06
Adjusted R-squared	0.4682	0.792	0.7795	0.995	0.9988	0.9413	0.7882	0.9775	0.7902	0.8932	0.8264	0.7574	0.9058	0.893	0.859	0.9532

**Figure 2:**
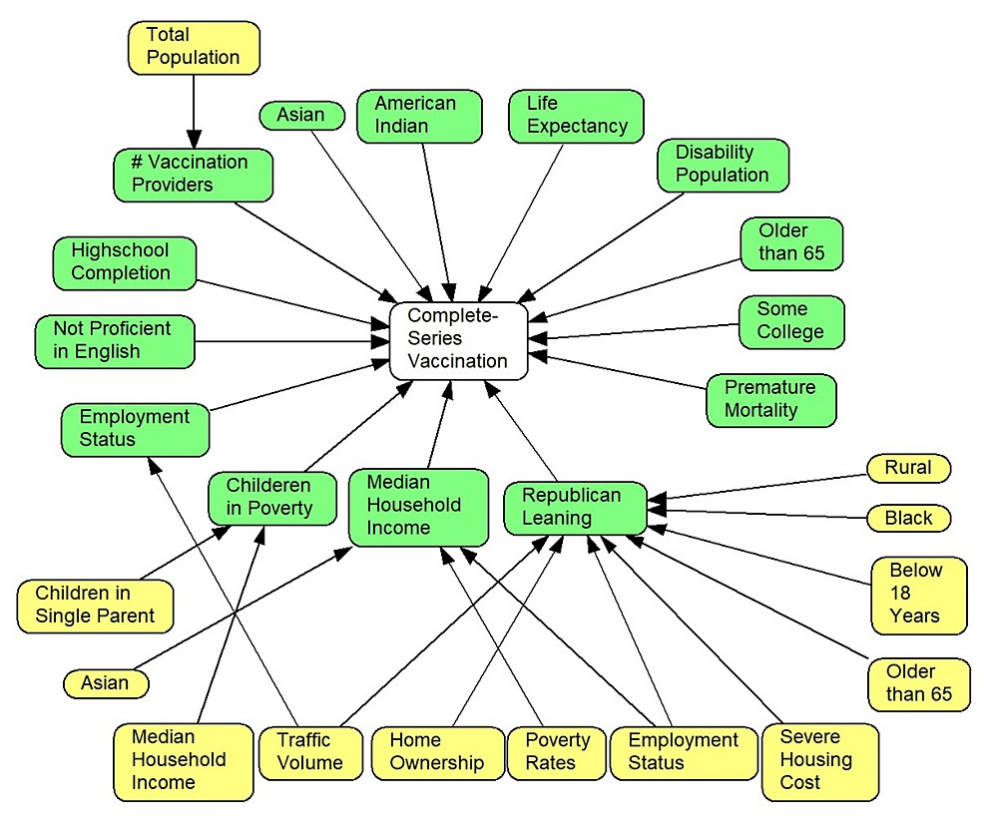
Social determinants, political leaning, and vaccination hesitancy Note: To enhance the comprehensibility of the network model, the associations between the same variable measured in two different time frames were purposely omitted. Specifically, the model displays variables measured at different timeframes, with the white-colored node representing the outcome of interest measured in April 2022. The green variables were measured in 2022, while the yellow variables were measured in 2020. Only variables that had a statistically significant association with vaccination hesitancy, indicated by a non-zero value, are depicted in the network.

Figure 2 depicts direct (green color measured in 2022) and indirect predictors (yellow color measured in 2020) of vaccination rates. A previous study by us demonstrated that a large number of factors predict vaccination rates [10].

In the previous study, the following 13 variables affected vaccination rates: (1) percent of residents with American Indians or Alaska Natives; (2) percent of Asian residents; (3) percent of senior residents (≥65 years). The percentage of Black or African American residents did not directly predict vaccination rates. (4) The higher the number of vaccination providers in the county, the higher the vaccination rates. Clearly, access mattered.

The following social determinants of illness affected vaccination rates: (5) percent of residents with high school completion; (6) percent of residents with some college; (7) percent of residents unemployed; (8) median household income; (9) percent of residents not proficient in English; and (10) percent of children in poverty.

The following health status variables affected vaccination rates: (11) average life expectancy of residents; (12) percent of residents with a disability; and (13) premature mortality rates.

These direct predictors of vaccination rate also mediated the effect of other factors, including (1) level of income inequality in the county, (2) traffic volume, (3) percent of residents with home ownership, (4) percent of children in single-parent homes, (5) percent of African American residents, (6) total population, (7) rural/urban settings, (8) severe housing costs, and (9) percent of residents employed.

Given these relationships, we examined if political affiliation was predictive of vaccine hesitancy. In the LASSO regression of vaccination rates on 36 2022-measured variables, 14 variables had a non-zero relationship with vaccination hesitancy, including the 13 variables listed above and political affiliation.

Discussion

Our analysis identified a variety of factors that affect vaccine hesitancy, as have a large number of previous studies. Education affected vaccine hesitancy. “Some college” and “high school completion” were positively associated with higher vaccination rates, as seen in other studies as well [11-14],. Race was also a factor in vaccine hesitancy; sometimes a direct predictor and other times an indirect predictor of vaccine hesitancy. Asian and American Indians or Alaska Natives were direct predictors of vaccination rates. Counties with a higher proportion of African Americans did not have more vaccine hesitancy, except indirectly through other mediators. This finding was not supported by the literature as cross-sectional studies have found the reverse [15-17]. One possible explanation of the divergence between our findings and the literature could be because vaccination hesitancy decreased more rapidly among African American population than the White over time [18], thus study findings may depend on when the study was done. Between the first and second year of the pandemic, racial and ethnic inequities in COVID-19 mortality also decreased [19]. Age remained a persistent direct predictor of higher vaccination rates. We found that the higher the proportion of the senior population (65 years or older) in the county, the higher the vaccination rates; other studies also supported this association [20,21]. Health status was a direct predictor of vaccination rates. The higher the “life expectancy,” the higher the vaccination rates; this finding has also been confirmed in the literature [22]. In terms of disability, a previous study also confirmed that adults with disabilities are less likely to have received COVID-19 vaccination than those without disabilities [23]. Socioeconomic factors also affect vaccination hesitancy. We found that the higher the “median house income,” the higher the vaccination rate. Others also found similar results [24]. We found that “unemployment” had a positive association with vaccination rates; others also found similar results [25-27]. In general, the current study confirmed that many factors affect vaccine hesitancy. The current study controlled these factors and examined the effect of political affiliation. We found that political affiliation affected vaccine hesitancy.

Before we discuss the impact of political affiliation on vaccination rate, it is important to point out that vaccination rates and political affiliations were affected by the same set of covariates. Counties that had a higher percentage of Republican supporters were also likely to be (1) rural, (2) White, and (3) had a higher percentage of residents over the age of 65 years or under 18 years. These counties were also more likely to have (4) lower unemployment rates, (5) lower levels of traffic volume, (6) lower levels of housing cost burden, and (7) lower levels of median house income. Additionally, higher poverty rates positively contributed to Democratic-leaning counties rather than Republican-leaning ones. The network model statistically controlled for these covariates and identified political affiliation as a mediator of vaccination rate. Seven variables (listed above) impacted the vaccination rate through increasing Republican political affiliation. These characteristics that increased the likelihood of supporting the Republican Party, with two exceptions (median household income and older than 65 years), did not directly affect vaccination rates. In short, party affiliation was a mediator of the effect of five variables that also increased party affiliation.

This study shows that party affiliation was not only an independent direct predictor of hesitancy but also a mediator of the effects of other variables. In statistics, when a variable is a mediator of the effects of other variables, it is generally interpreted that it shows the mechanism through which the indirect variables affect the outcome. There are many ways in which party affiliation may have acted as the mechanism leading to lower vaccination rates. One study has suggested that political affiliation leads to media choices that could affect vaccine hesitancy [28]. Another study has suggested that the Republican Party’s economic framing of vaccination could have led to hesitancy [29]. Still, another way that the Republican Party might have affected vaccine hesitancy is by making it socially acceptable to not vaccinate [30,31], or altering perceptions of risk [32]. Yet another study shows that party members were responding to the cues that Republican Party leaders were providing [33]. Our study does not describe how the Republican Party played an organizing role in vaccine hesitancy but it does show that it did so.

Conclusions

Political affiliation plays an important direct, and mediating, role in reducing vaccination rates. The direct effect of political affiliations on vaccine hesitancy remained despite the use of a variety of alternative explanations. It also appears that these affiliations played an organizing role and mediated the effects of a variety of other variables. This study, and other investigators, suggest that interventions and public health policies should be designed to specifically address the impact of political affiliation on vaccine hesitancy [6].
